# Cognitive and Psychological Outcomes Following Pediatric Cardiac Arrest

**DOI:** 10.3389/fped.2022.780251

**Published:** 2022-02-09

**Authors:** Nathan A. Huebschmann, Nathan E. Cook, Sarah Murphy, Grant L. Iverson

**Affiliations:** ^1^Department of Physical Medicine and Rehabilitation, Spaulding Rehabilitation Hospital, Charlestown, MA, United States; ^2^Department of Physical Medicine and Rehabilitation, Harvard Medical School, Boston, MA, United States; ^3^New York University Grossman School of Medicine, New York, NY, United States; ^4^Division of Pediatric Critical Care, MassGeneral Hospital for Children, Boston, MA, United States; ^5^Department of Pediatrics, Harvard Medical School, Boston, MA, United States; ^6^Spaulding Research Institute, Charlestown, MA, United States

**Keywords:** pediatric cardiac arrest, cognitive outcomes, adaptive behavior, psychological health, family functioning, rehabilitation needs, academic accommodations

## Abstract

Cardiac arrest is a rare event in children and adolescents. Those who survive may experience a range of outcomes, from good functional recovery to severe and permanent disability. Many children experience long-term cognitive impairment, including deficits in attention, language, memory, and executive functioning. Deficits in adaptive behavior, such as motor functioning, communication, and daily living skills, have also been reported. These children have a wide range of neurological outcomes, with some experiencing specific deficits such as aphasia, apraxia, and sensorimotor deficits. Some children may experience emotional and psychological difficulties, although many do not, and more research is needed in this area. The burden of pediatric cardiac arrest on the child's family and caregivers can be substantial. This narrative review summarizes current research regarding the cognitive and psychological outcomes following pediatric cardiac arrest, identifies areas for future research, and discusses the needs of these children for rehabilitation services and academic accommodations.

## Introduction

Cardiac arrest is relatively rare in childhood. The incidence of nontraumatic out-of-hospital arrest is about 8 per 100,000 person years (1, 2) and a scientific statement from the American Heart Association estimates that >7,000 children in the United States experience out-of-hospital cardiac arrest each year (3, 4). Critically ill children, however, are at much greater risk. A multicenter prospective observational study of children followed from intensive care unit admission to hospital discharge reported that, in a cohort of 10,078 children, 139 (1.4%) received cardiopulmonary resuscitation (5). Pre-existing cardiac disease is one risk factor for pediatric in-hospital cardiac arrest, which can result from the progression of cardiac, respiratory, neurologic, gastrointestinal, or neoplastic disease processes (5–7). Cardiac arrest suffered outside the hospital most often has a respiratory etiology such as drowning/asphyxia or progressive respiratory failure (6, 8). Rates of survival to hospital discharge range from 22 to 54% for in-hospital cardiac arrest (9–13) but have been reported to be as low as only 6.4 to 11.4% for out-of-hospital cardiac arrest (1, 2, 8). The neurological morbidity is difficult to assess, and outcomes vary widely from survival in a vegetative state to apparent swift and seemingly full clinical recovery (6). Out-of-hospital cardiac arrest is associated with worse neurological outcomes than in-hospital arrest at both discharge (6) and one year follow-up (14). The neurobiological mechanisms underlying neurological deficits include hypoxic-ischemic injury (15), but could also be related to brain injury upon reperfusion (secondary to excitotoxicity, calcium accumulation, protease activation, and formation of reactive oxygen and nitrogen species), neuronal damage due to a combination of apoptosis, autophagy and necrosis, and inflammation (7) and underlying and associated disease processes that lead to cardiopulmonary compromise.

Although survivors of pediatric cardiac arrest may have broadly favorable neurological and functional outcomes, many survive with measurable neurologic and functional deficits (9, 14, 16–19). The purpose of this review is to summarize current understanding of cognitive, behavioral, and psychological outcomes of children and adolescents who survive cardiac arrest. We conducted targeted literature reviews for each topic in this narrative review. This review concisely summarizes and integrates findings from studies that have measured neuropsychological and psychosocial outcome, broadly defined. We draw attention to the potential long-term impairments in neuropsychological functioning, limits in current understanding, as well as the potential follow-up treatment and rehabilitation needs.

## Adaptive Behavior and Functioning

Broadly speaking, a child's adaptive behavior and functioning refer to the skills required to function well in one's environment and everyday life. Adaptive functioning in children who have suffered cardiac arrest has been assessed in studies using the Vineland Adaptive Behavior Scales-Second Edition (VABS-II; or the third edition, VABS-III) (20), a comprehensive parent-report measure that evaluates the domains of motor skills, communication, daily living, and socialization (21–30). Many studies have found impairments in adaptive functioning (22, 24, 25, 27–29), motor functioning (22, 24, 27–29), communication (22, 27–29), and daily living skills (22, 24, 27–29), when assessed at various time points, including 3 and 12 months following cardiac arrest. These impairments were typically determined when compared to parent ratings of their child's baseline, or pre-cardiac arrest functioning. In studies performed to date, impairments in adaptive functioning are less commonly detected than deficits in cognitive functioning (27, 30); although cognitive impairments may be diagnosed in up to half of survivors, studies have reported that the majority of children who survive cardiac arrest will score within broadly normal limits of adaptive functioning based on parent ratings (24, 25, 27, 28).

**Figure 1:**
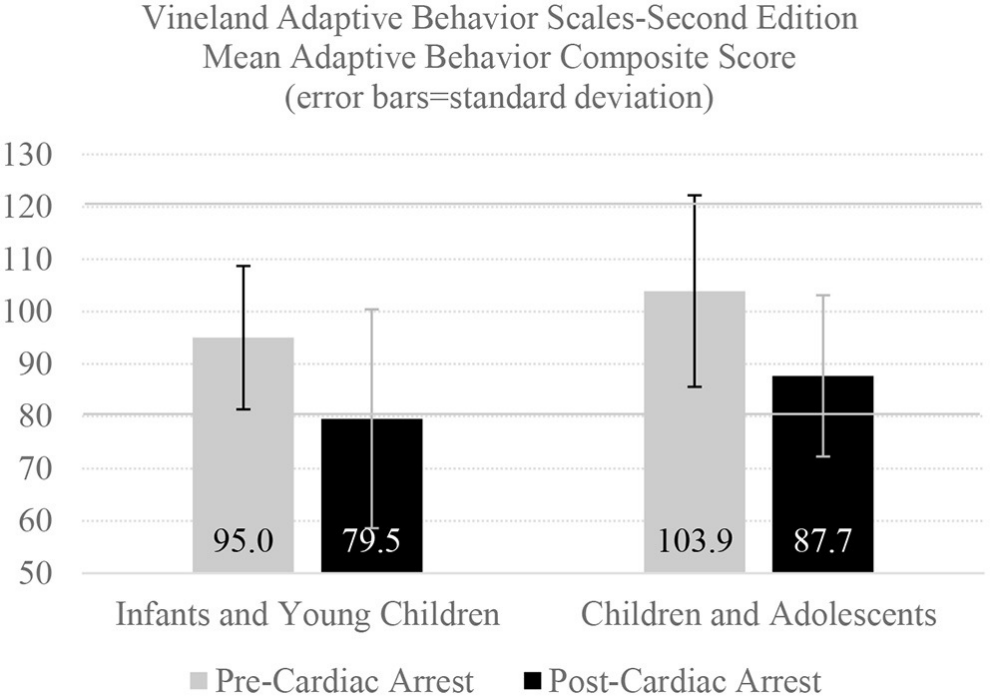
One-year adaptive behavior outcomes following cardiac arrest. There were 146 infants and young children who were enrolled in one of two clinical trials for whom pre-arrest and one-year post-arrest adaptive behavior ratings were completed by parents (median age = 0.5 years, interquartile range = 0.2–1.7, all under the age of 5 at the time of cardiac arrest), as presented in the online supplement (30). There were 49 children and adolescents with pre- and post-arrest adaptive behavior ratings (median age = 13.8 years, interquartile range = 9.2–16.2 at the time of cardiac arrest). The Adaptive Behavior Composite Score has a normative mean of 100 and a standard deviation of 15, so 90% of infants, children, and adolescents in the normative sample would be expected to obtain scores between 80 and 120, demarcated with a solid horizontal line. This figure uses means and SDs presented in Slomine et al. (30).

However, a sizeable percentage of children with normal premorbid adaptive functioning, including as many as half of those who survive an out-of-hospital cardiac arrest (29), exhibit significant adaptive functioning deficits at 1 year following arrest (29, 30). This is illustrated in Figure 1 (30), which was produced from means and standard deviations provided in the supplementary online content of the source article (30). A large percentage will have very low adaptive functioning, below the 10^th^ percentile normatively (30).

Children who suffer an out-of-hospital cardiac arrest have, on average, worse adaptive functioning 1 year later (14, 22). Gross neurological functioning at hospital discharge, as assessed by the Pediatric Cerebral Performance Category, is strongly associated with VABS-II score at both 3 and 12 months post cardiac arrest and VABS-II scores tend to remain fairly stable between 3 months and 12 months after the event (31, 32). Extracorporeal membrane oxygenation (i.e., ECMO, an artificial lung), preexisting gastrointestinal conditions, and higher blood lactate levels following cardiac arrest have been associated with more adaptive behavior difficulties at 1 year (25, 33).

## Cognitive Functioning

Many children who survive cardiac arrest have persistent or long-term deficits in cognitive functioning (14, 22, 24, 26–30, 34–37), including deficits in attention (30, 38), language (14, 36), memory (30, 37, 39), executive functioning (26, 30), and overall intellectual functioning (26, 28, 34, 37). These cognitive deficits can be severe (22, 28, 29, 36). Impaired or severely impaired long-term cognitive functioning is more prevalent in children who suffer out-of-hospital cardiac arrest (24, 27, 28, 34). Amongst a subgroup of children with broadly normal adaptive functioning 1 year following either out-of-hospital or in-hospital cardiac arrest, approximately 25% still scored in the impaired or severely impaired range on intellectual testing (30). In children with broadly normal premorbid adaptive functioning, neurologic status, as assessed by a neurologic examination 1 year following cardiac arrest, has been shown to be significantly correlated with cognitive functioning assessed by neuropsychological testing (14). One study found that older age at hospital admission is a significant predictor of parent-reported attention problems at long-term follow-up 2–11 years after cardiac arrest (38). Additionally, duration of cardiac arrest, age <6 months, and number of comorbid medical risk factors such as congenital heart disease and low weight (≤ 5^th^ percentile) have been shown to be significantly and negatively correlated with composite scores of intelligence when assessed 1 year or more following cardiac arrest (26).

**Figure 2:**
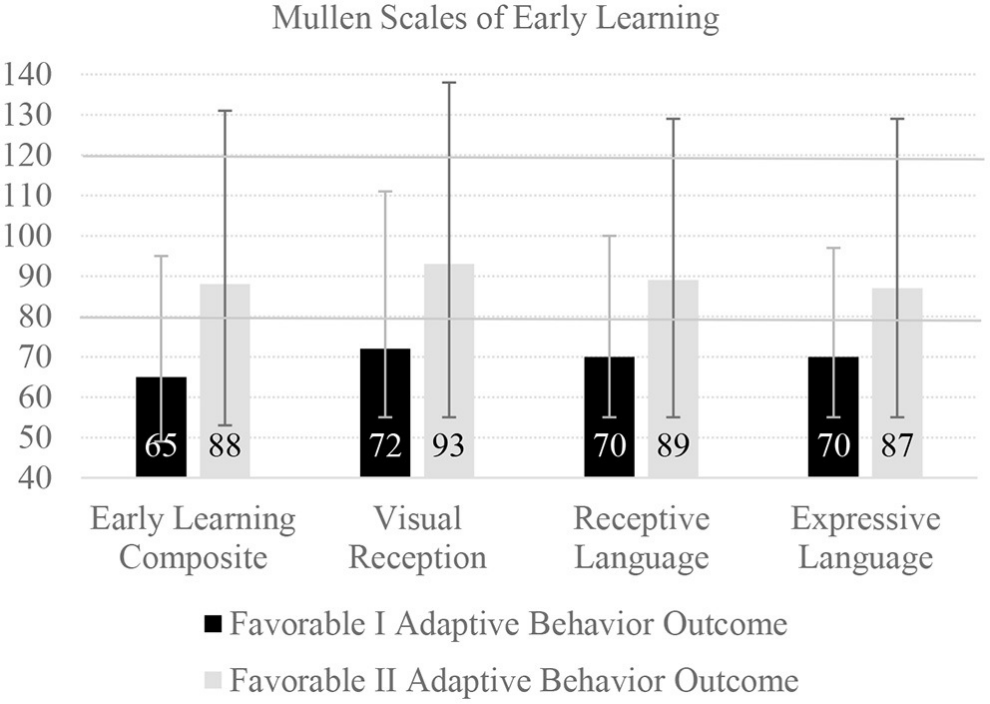
One-year neuropsychological outcome following cardiac arrest in young children. There were 119 children in this study with outcome data at one-year post cardiac arrest (median age = 1.6, interquartile range = 1.2–3.1), with parent-rated mean pre cardiac arrest adaptive behavior composite scores of 95.2 (SD = 14.2) (30). There were 24 children in the Favorable I group who had adaptive behavior composite scores between 70 and 84, and 55 in the Favorable II group who had adaptive behavior composite scores of 85 or greater. The bars represent mean scores and the error lines represent the range of scores. The Mullen Scales are presented with a normative mean of 100 and a standard deviation of 15, so 90% of children in the normative sample would be expected to obtain scores between 80 and 120, demarcated with a solid horizontal line. The Early Learning Composite is an overall measure of functioning. The Visual Reception composite measures the ability to understand symbols and pictures, and spatial recognition of objects. This figure uses means and ranges presented in Slomine et al. (30).

Secondary analyses of two major clinical trials (30), focused on one-year neuropsychological outcome in pediatric patients who were comatose following both in- and out-of-hospital cardiac arrest, illustrate several important issues. Global cognitive impairment was found in 55.6% of the 117 young children (age 5 or younger), most of whom were between the ages of 1 and 3 at the time of assessment. One-year neuropsychological outcomes are illustrated in Figure 2, which was produced from the means and ranges provided in Table 4 of the source article (30). Importantly, these children were conceptualized as having “favorable” functional outcome based on their parents' ratings of their daily functioning on the Vineland Adaptive Behavior Rating Scales-Second Edition. The research team defined favorable liberally as any composite score that was within two standard deviations of the normative mean (30). Two favorable outcome groups were created, those with an adaptive behavior composite score between 1 and 2 standard deviations below the mean (i.e., 70–84) and those who had a composite score 1 standard deviation below the mean or higher (i.e., 85 or greater). As seen in Figure 2, functional outcome was associated with global cognitive function and children with below average functional abilities had pronounced deficits in early learning, visual perception, receptive language, and expressive language. A very large percentage scored below the broadly normal range (i.e., below the 10^th^ percentile, a standard score of 80, illustrated be the lower horizontal line in the figure). Clearly, these children should be identified and provided with occupational therapy and speech and language services prior to entering the school system.

**Figure 3:**
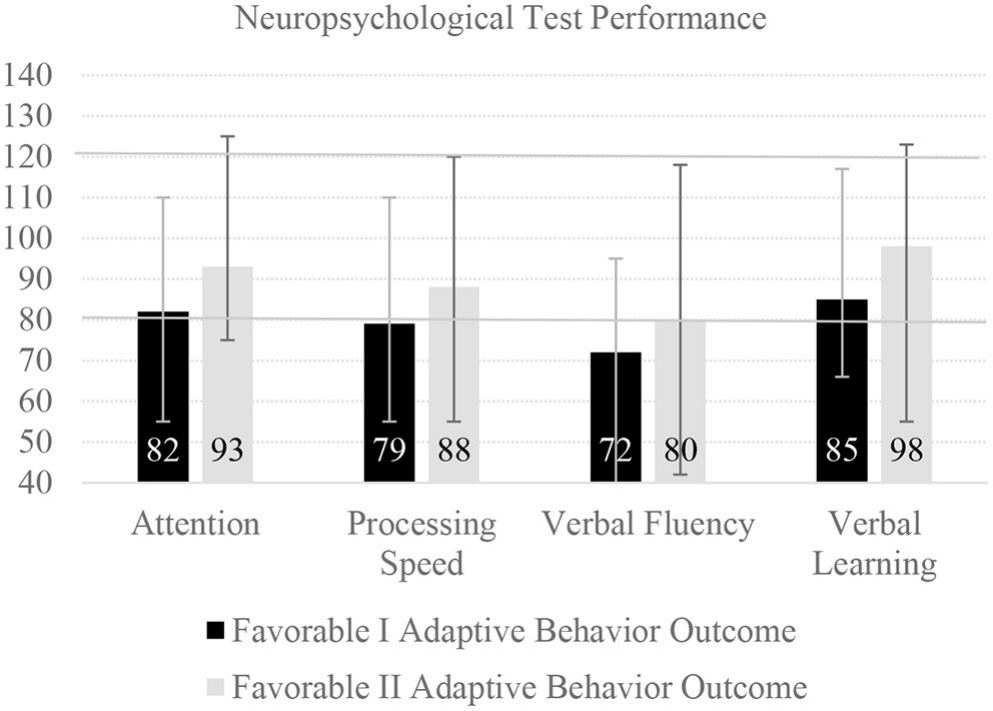
One-year neuropsychological outcome following cardiac arrest in children and adolescents. There were 41 youth in this study with outcome data at one-year post cardiac arrest (median age = 14.3, interquartile range = 10.3–16.4), with parent-rated mean pre cardiac arrest adaptive behavior composite scores of 103.9 (SD = 19.2) (30). There were 10 youth in the Favorable I group who had adaptive behavior composite scores between 70 and 84, and 25 in the Favorable II group who had adaptive behavior composite scores of 85 or greater. The bars represent mean scores and the error lines represent the range of scores. The neuropsychological tests were converted to have a normative mean of 100 and a standard deviation of 15, so 90% of youth in the normative sample would be expected to obtain scores between 80 and 120, demarcated with a solid horizontal line. The tests included were as follows: Attention (Digit Span), Processing Speed (Coding), Verbal Fluency (Controlled Oral Word Association Test), and Verbal Learning (California Verbal Learning Test-Children's Edition Trials 1–5 Total Score). This figure uses means and ranges presented in Slomine et al. (30).

Forty-one older children and adolescents were analyzed separately, most of whom were between the ages of 10 and 16 at the time of assessment (30). One-year neuropsychological outcomes are illustrated in Figure 3, which was also produced from the means and ranges provided in Table 4 of the source article (30). These children and adolescents were also dichotomized as having “favorable” outcome based on their parents' ratings of their daily functioning, on the Vineland Adaptive Behavior Rating Scales-Second Edition—similar to the young children presented in Figure 2. As seen in Figure 3, 15.0% had global cognitive deficit, but specific deficits were commonly identified, especially among those with below average functional abilities, including deficits in attention, processing speed, verbal fluency, and verbal learning. Likewise, a very large percentage scored below the broadly normal range (i.e., below the 10^th^ percentile, a standard score of 80, illustrated by the lower horizontal line in the figure). Clearly, these youth should be identified and provided with services to promote the best possible academic outcomes.

## Emotional Problems, Psychological Stress, and Family Functioning

There has been limited research regarding psychological and emotional health outcomes following pediatric cardiac arrest. Some children experience difficulties with anxiety, depression, and behavioral problems (26, 38, 40). A Dutch study examined 52 children and adolescents 2–11 years post cardiac arrest (38). For the pre-school-aged children (age 5 and younger), 42% scored higher than expected for the total number of problems on the Child Behavior Checklist. The scales with the largest effect sizes were being withdrawn (Cohen's d = 0.64) and attention problems (d = 0.74). For school aged children and adolescents, only 19% scored higher than expected for the total number of problems on the Child Behavior Checklist. The scales with the largest effect sizes were somatic complaints (Cohen's d = 0.90; particularly headache and abdominal pain) and attention problems (d = 0.70). Unexpectedly, neither the pre-school aged children or school aged children had elevations on the anxiety/depression scale (38). The authors speculated that the reasonably favorable emotional outcomes in the youth could relate, in part, to post-traumatic growth. Post-traumatic growth can be conceptualized as “the experience of positive change as a result of the struggle with highly challenging life crises” (41). Another psychosocial concept relating to resilience in family systems is called “response shift” (42), whereby people change their evaluation of quality of life in response to changes in their internal standards, values, and conceptualization of quality of life when they are faced with a life threatening or chronic disease.

The broader literature relating to pediatric heart disease and critical illness offers some insight into possible emotional health problems for children who experience cardiac arrest. Research in children who have congenital heart defects (43) or who undergo cardiac surgery (44) indicates that some will experience considerable traumatic stress and even meet diagnostic criteria for posttraumatic stress disorder (PTSD). Furthermore, a recent study found that in a sample of patients aged 8–21 years assessed a mean of 2.6 years following cardioverter defibrillator implant, 25% met the clinical cutoff for anxiety and 19% met the clinical cutoff for depression on self-report measures (45). Importantly, there is potential for psychological treatment to address post-traumatic stress and reduce anxiety in children following cardiac arrest. A recent review (46) examined 16 studies of interventions for pediatric medical traumatic stress, defined as a “set of psychological and physiological responses of children and their families to pain, injury, serious illness, medical procedures, and invasive or frightening treatment experiences” (47). The authors concluded that interventions including caregiver involvement and cognitive behavior therapy principles, especially those that are online, self-guided, and time-limited, show promise for reducing post-traumatic stress symptoms in patients and caregivers (46). Another psychological consideration is death anxiety, described as a state of worry, discomfort, or fear related to dying due to a real or imagined threat to one's existence (48). Death anxiety has been observed in children with terminal illnesses, such as cancer (49, 50), and survivors of liver transplants (51). We could find no prior studies that have examined death anxiety in survivors of pediatric cardiac arrest. It is reasonable to suspect that death anxiety is a domain of clinical and research interest in this population.

**Figure 4:**
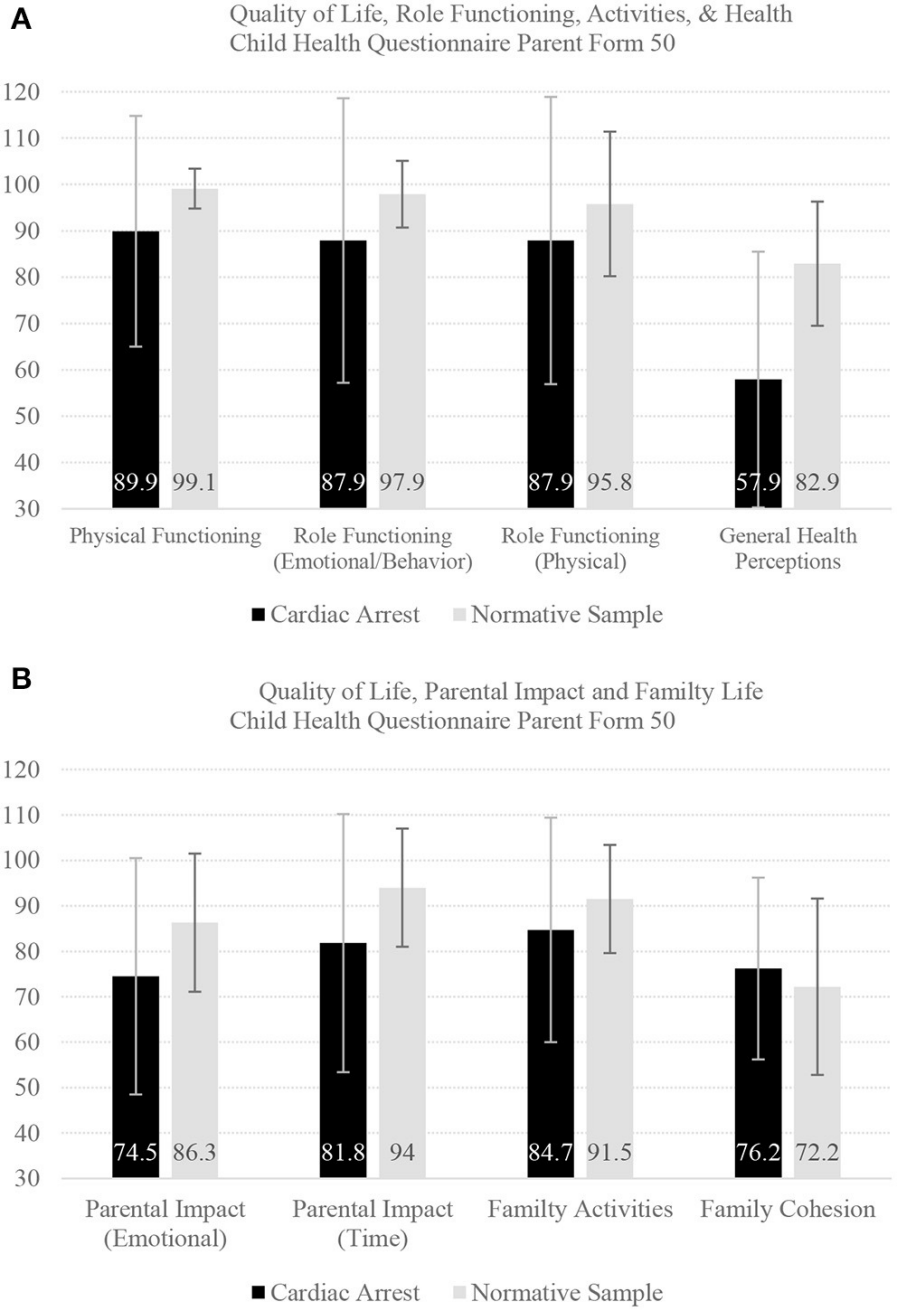
Quality of life outcomes in children and adolescents. There were 33 children and adolescents followed a median of 5.6 years following either in hospital or out of hospital cardiac arrest using the Child Health Questionnaire (53). They were compared to 353 youth in a normative sample. The bars represent mean scores and the error lines represent the standard deviation. Child Health Questionnaire Parent Form 50 Questions, differences between groups in Cohen's d effect sizes: Physical Functioning (Cohen's d = 1.11): limitations in playing sports, recreational activities, walking, or self care; Role Functioning (Emotional/Behavior Limitations) (d = 0.89): school work or social life limited by emotional or behavioral difficulties; Role Functioning (Physical Limitations) (d = 0.45): school work or social life limited by physical health problems; General Health Perceptions (d = 1.66): child seems less healthy than other children; Parental Impact (Emotional) (d = 0.72): parental emotional concern about child; Parental Impact (Time) (d = 0.82): parents being limited in the amount of time for their own needs because of child; Family Activities (d = 0.51): child's health or behavior limits family activities; and Family Cohesion (d = 0.21): family's ability to get along with one another. This figure uses mean scores and standard deviations presented in Van Zellem et al. (53).

Pediatric cardiac arrest can have major effects on the family system. Parents experience a considerable degree of caregiver burden, including anxiety or worry about their child, limited time for personal needs, and interference with family activities due to their child's health or behavior, three and 12 months following cardiac arrest in their child (23, 52). Additionally, worse neurobehavioral functioning in children at 3 months has been associated with greater caregiver burden at 12 months following cardiac arrest (23, 52). One Dutch study examined health-related quality of life in children and adolescents at a median of 5.6 years following either in-hospital or out-of-hospital cardiac arrest using the Child Health Questionnaire (53). The results of that study are illustrated in Figure 4, which was produced from means and standard deviations provided in Table 3 of the source article (53). Parents reported a variety of health problems in these youth, and that their children's activities were limited by these health problems. The parents themselves experienced emotional health concerns associated with their child's health and functioning.

## Neurological Problems and Rehabilitation Needs

Children who survive cardiac arrest may experience a wide range of neurological deficits. The Pediatric Cerebral Performance Category is used to measure gross neurological function and consciousness in children following cardiac arrest (54) at hospital discharge (5, 6, 9, 10, 13, 14, 16–19, 55–57) and follow-up periods ranging from 1 month to several years after cardiac arrest (10, 55, 57, 58). Gross neurological functioning varies from normal to a vegetative state. Large proportions of surviving children have been reported as having “favorable” or “good” neurological outcomes (5, 9, 16–19, 59, 60). However, “favorable” or “good” status was broadly defined and included mild (i.e., PCPC = 1–2) and/or moderate (i.e., PCPC = 1–3) disability, or no change in neurological status from admission. Specific neurological deficits such as aphasia and apraxia have been reported following cardiac arrest in children as well (39). On neurologic examination 1 year following cardiac arrest, over 50% of children have been found to have at least mild neurological impairments, including sensorimotor deficits, deficits in language production and comprehension, or other motor or sensory deficits (including cranial nerve deficits), with about 30% having severe or profound impairments (14, 24).

**Figure 5:**
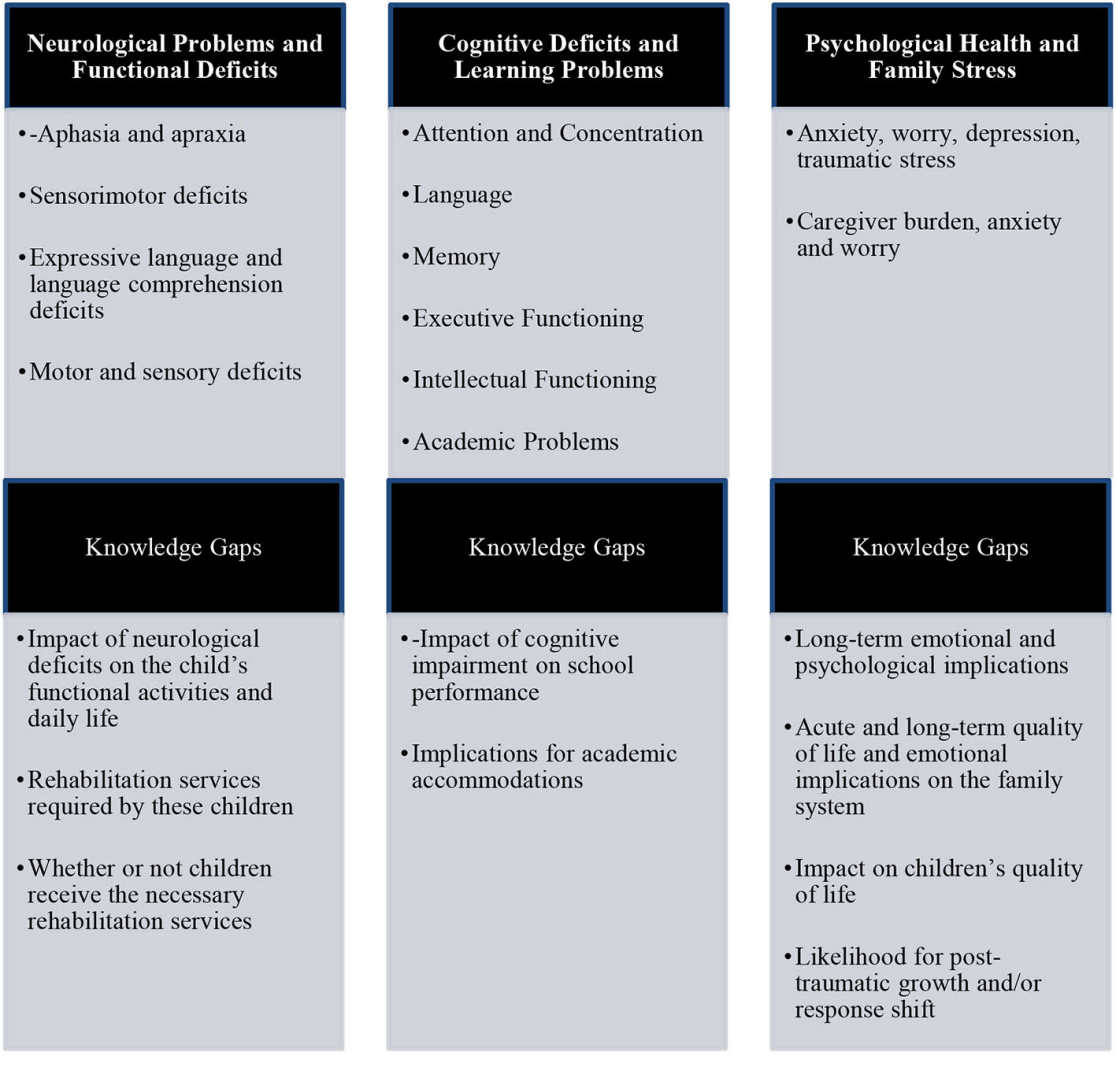
Treatment and rehabilitation needs of children following cardiac arrest. Post-traumatic growth can be conceptualized as experiencing positive outcomes as a result of struggling with highly challenging life crises (41). Response shift (42) refers to a psychosocial process whereby people change their evaluation of quality of life in response to changes in their internal standards, values, and conceptualization of quality of life when they are faced with a life threatening or chronic disease.

Clearly, many of these children will have functional difficulties in their daily life and many will likely experience disrupted cognitive or social development for years to come. Therefore, children who experience cardiac arrest may require rehabilitation services, including speech, physical, and occupational therapy, and to benefit from long-term monitoring of their social, emotional, cognitive, and academic development (4) (see Figure 5).

## Academic Accommodations

Many children who experience cardiac arrest will require academic accommodations upon their return to school. These accommodations might be for ongoing physical, emotional, cognitive, learning, and/or medical problems. Some children might have had accommodations in place prior to their cardiac arrest due to a chronic medical condition. Physicians can provide documentation to support and advocate for academic accommodations, such as by documenting a child's diagnoses and qualifying health conditions and recommending for the school what specific services and supports a child would likely benefit from. In the United States educational system, there are two mechanisms by which a student may receive accommodations: 504 plans and Individualized Education Programs (IEP). 504 plans require a less formal process. They are described in Table 1. An IEP sets out individualized special education and specific services designed to address a child's unique academic needs (61), as described in Table 2. Similar support programs exist in other countries to aid children requiring accommodations. There is a lack of research examining the implementation and effects of academic accommodations for children following cardiac arrest. Therefore, while it is apparent that children who experience cardiac arrest will likely require accommodations due to the sequelae described above, little is known about their effectiveness. This is an important area of future research with implications for children and their families.

**Table 1:** 504 Plans.

**Law:** From Section 504 of the Rehabilitation Act of 1973, a Federal civil rights law, which legislates that children with disabilities receive accommodations allowing them access to the learning environment.
**Documentation:** To initiate a 504 plan, documentation of a disability by a physician, psychologist, or other qualified healthcare provider is generally sufficient.
**Plan:** School usually provide these plans in writing (although this is not required). This plan documents special services that will be provided, and changes to the learning environment, to enable students to learn alongside their peers. The 504 plan is broader and more inclusive than an IEP in that it includes any form of disability that interferes with the child's ability to learn in a general classroom.
**Transfer of Plan:** A 504 plan can transfer from high school to college and even into the workplace.

**Table 2:** Individual education plans.

**Law:** Individual Education Plans (IEPs) are legislated by a Federal law for children with disabilities entitled the Individuals with Disabilities Education Act (IDEA).
**Eligibility:** To be eligible for an IEP, a child must have (i) have a specific, qualifying disability (e.g., learning disability, attention-deficit/hyperactivity disorder, autism spectrum disorder, emotional health condition, speech or language impairment), that (ii) adversely affects the child's ability to benefit from the general education curriculum provided by the school and make effective academic progress.
**Process:** IEPs are created by a team including a parent, at least one general education teacher, at least one special education teacher, a school psychologist or other specialist who can interpret evaluation results, and a representative with authority over special education services.
**Documentation:** IEPs are always documented in writing and approved by parents and school personnel. The IEP formally documents (i) the child's present level of functioning, and how the child is doing in school; (ii) annual education goals and how they will be tracked; (iii) specific accommodations (changes to the learning environment), modifications (changes to what the child is expected to learn) and special education services (e.g., speech, physical, or occupational therapy) to be provided; (iv) the timing of all accommodations, modifications, and services (e.g., start, frequency, duration); (v) how the child will participate in standardized testing; and (vi) how the child will be included in the general education environment (61).
**Transfer of Plan:** IEPs do not transfer to college or the workplace.

## Conclusions and Future Directions

Outcome among survivors of pediatric cardiac arrest varies broadly from very good functional recovery to permanent and severe disability. Many of these children will require rehabilitation services, such as speech and language, physical therapy, and occupational therapy. These children and adolescents experience varying degrees of impairment in intellectual and cognitive functioning. More research is needed to understand cognitive deficits in the weeks and months following pediatric cardiac arrest, the association of acute and subacute cognitive weaknesses with short- and longer-term functional outcomes (e.g., school performance), and the course of cognitive difficulties over time. Some of the studies to date have been conducted with samples of infants and toddlers—and long-term follow-up during early childhood and adolescence would be helpful for understanding their treatment, rehabilitation, social-emotional, and educational needs.

Some children and families do very well after this life-altering event. However, some of these youth experience depression, anxiety, traumatic stress, and behavior problems—and associated parental mental health difficulties can exacerbate these difficulties in the children and create difficulties within the family system. More research is needed to understand the nature and extent of psychological and emotional health problems in these children and their families, with the goal of informing timely and effective treatment. Most children will require short-term or long-term academic accommodations for physical, cognitive, emotional, or medical problems, and treating physicians can play an important role in initiating these accommodations.

## Author Contributions

NH reviewed and summarized the literature, wrote drafts of sections of the manuscript, edited drafts of the manuscript, and approved the final version for submission. NC helped conceptualize the review, edited drafts of the manuscript, and approved the final version for submission. SM edited drafts of the manuscript and approved the final version for submission. GI conceptualized the review, wrote sections of the manuscript, edited drafts of the manuscript, and approved the final version for submission. All authors contributed to the article and approved the submitted version.

## Conflict of Interest

The authors declare that the research was conducted in the absence of any commercial or financial relationships that could be construed as a potential conflict of interest.

## Publisher's Note

All claims expressed in this article are solely those of the authors and do not necessarily represent those of their affiliated organizations, or those of the publisher, the editors and the reviewers. Any product that may be evaluated in this article, or claim that may be made by its manufacturer, is not guaranteed or endorsed by the publisher.
